# Work-life balance and employee commitment: mediating effect of job satisfaction

**DOI:** 10.3389/fpsyg.2024.1349555

**Published:** 2024-05-30

**Authors:** Henry Egbezien Inegbedion

**Affiliations:** Department of Business Administration, Bowen University, Iwo, Nigeria

**Keywords:** employee commitment, job satisfaction, work-life balance, family, health 1

## Abstract

**Introduction:**

Work is very important to man and work experiences have implications on other aspects of human life. The major essence of work is to obtain resources to optimize other domains of life. This demands the need to strike a balance. Flowing from the positive and negative effect of the Spill over theory, the study investigated the influence of work-life balance (WLB) on employee job commitment using JS as the mediating variable.

**Methods:**

A cross-sectional survey design elicited the desired data from 344 employees in 4 Universities and 4 multinational companies. Path diagram analysis served as the data analysis technique.

**Results/discussion:**

The results show that all the family and religious lives have statistically significant negative influences on employee commitment (EMPC) while leisure and health have statistically significant positive influences on EMPC. The study thus, makes theoretical contributions.

## 1 Introduction

An individual’s inability to balance work-life often precipitates emotional and psychological problems that may infringe on his JS and ultimately his commitment and performance. A very important question that every worker needs to answer is; “do we work to live or live to work? An appropriate answer to this question will predispose the worker toward a rational consideration of WLB. (WLB) There is empirical evidence that an employee’s two most important domains are his/her work and the home (or family) and the experience at work or home almost always defines the individual’s emotional stability and/or happiness (Greenhaus et al., 2003; Wambui et al., 2017). It is reasonable to argue that an individual worker realizes himself through the work he does as well as through his capacity for self-actualization through the attainment of the very zenith of the career of his choice. However, only an emotionally stable and healthy person can have the right frame of mind for the required level of commitment for meaningful contributions to the attainment of organizational goals. To this end, an ideal self-actualization will contribute to the integration of both the work and basic aspects of life (family, personal life, community service) (Wambui et al., 2017). Securing employment in an organization should not be at the expense of an individual’s life; rather; securing a job demands that the individual maintain equilibrium by striking a balance between both work and life. Striking an equilibrium between the commitment to work and life is required for a healthy life since only a healthy person can work well.

Because of WLB’s importance to employee fulfillment, many scholars have investigated the research problem in recent times. Some of the studies include WLB and its importance of work–family and work–Health Balance (Gragnano et al., 2020), the influence of WLB and JS on the job performance of the Employees of SMEs moderated by family-supportive supervisor behaviors” (Susanto et al., 2022). The others are the influence of WLB on the self-reported health of some working adults in Europe using comparative analysis based on gender and welfare state regime (Mensah and Adjei, 2020) and WLB from the organizational context (Delecta, 2011). In addition, WLB and firm innovativeness based on male and female bosses’ strategies (Shouman et al., 2022), determinants of WLB among teachers of a government school in Sri Lanka (Umma and Zahana, 2020). Examination of the extent to which WLB, health and well-being in European welfare states can be a balancing act (Lunau et al., 2019) and the influence of JS and WLB on the job performance of female Nurses, and lastly, the influence of WLB on JS (Abdullah et al., 2022). Other empirical studies on WLB include Agha et al. (2017), Qureshi et al. (2019), Berglund et al. (2020), Ahmad and Raja (2021), Pehlivanoğlu et al. (2022), Bocean et al. (2023), Cheung G. W. et al. (2023), and Gupta and Agrawal (2023). Most of the studies used cross-sectional surveys and literature reviews as their designs. Nevertheless, not many studies have tested the mediating effect of job satisfaction on the relationship between work-life balance and employee commitment. It is also interesting to note that despite the robust literature on WLB, there is scantly any that examined how WLB relates with EMPC, neither is there any that incorporates family, religion, leisure and health in a singly framework to examine WLB. Most of the studies omitted religion (Delecta, 2011; Mas-Machuca et al., 2016; Hsu et al., 2019; Lunau et al., 2019; Gragnano et al., 2020; Mensah and Adjei, 2020; Otuya and Andeyo, 2020; Khateeb, 2021; Abdullah et al., 2022; Wijaya and Suwandana, 2022; Boakye et al., 2023; Thilagavathy and Geetha, 2023). Others omitted leisure (Delecta, 2011; Lunau et al., 2019; Mensah and Adjei, 2020; Otuya and Andeyo, 2020; Susanto et al., 2022; Wijaya and Suwandana, 2022) while Lunau et al. (2019) omitted family. Given the importance of religion and leisure to life in Nigeria, the neglect of religion and family in the consideration of work-life balance is a significant constraint. This study seeks to fill these gaps.

## 2 Literature review

WLB is concerned with the deliberate equilibration of the management of a person’s personal and work life. An individual attains a WLB when he feels at ease in the management of the obligations that have to do with his/her family and job. There is a need for individuals to make efforts to equilibrate personal time with the time spent on paid work and unpaid work to permit the smooth running of employment and life. Furthermore, WLB is the power that can facilitate the enhancement of productivity and efficiency in various aspects of the work and life of an individual employee to enable the making of good decisions (Abdullah et al., 2022). A similar concept is the work-family balance, which concerns the proper integration of an individual’s work and family life to permit his active engagement in both domains. It refers to a balanced work and family life. WLB requires that an individual separates his personal and professional life and accords each domain with the desired attention to prevent attention on one domain from having negative effects on the other. If an individual gives more attention to one than the other domain receives, the other domain will likely experience negative consequences because of the inverse relationship between both domains. This underscores the need for WLB.

WLB concerns the realization of experiences of fulfillment in an employee’s different aspects of life that require different types of resources such as energy, time and commitment, which often spread across all the domains (Kirchmeyer, 2000). Work is an aspect of a man’s life and not the entirety of his life. Other concerns provide him with emotional and psychological stability, especially his family. While it is common for people to compare WLB to work-family balance, it is pertinent to note that WLB consists of many roles that an individual assumes in society (Khateeb, 2021). In the modern context, WLB is increasingly becoming challenging, owing to the difficulty in equilibrating work with other domains of life. Added to the constraint posed to individual employees, critical stakeholders across the board are now taking up WLB as an important concept and are conscious of the need to mitigate the conflict that confronts the work and life territories through the formulation of policies and appropriate plans of action.

### 2.1 Theoretical framework

#### 2.1.1 The spill-over theory

The spill-over model assumes the addition of experiences from the work environment to non-work environment in a way that people perceive the social experience at the environment of work and non-work environment for an individual is effectively boundary-less (Parker and Scott, 1971). From a theoretical viewpoint, spill-over is characterized as positive and negative. The major thrust of positive spill-over is that fulfillment and attainment in one domain are a consequence of positive experiences in another environment (Vijayakumar and Janakiram, 2017). However, the negative spill-over, also known as dissimilar or incongruent in literature, states that work and non-work spheres relate inversely and antithetically (Staines, 1980; Khateeb, 2021). We can also categorized spill-over as vertical and horizontal. The former is the effect that one environment of life has on the neighboring environment such as the affect that JS may have on private life. The latter, which we express, using the realm of hierarchy, is the hierarchical organization of domains of life such as job, family, leisure, and religion, among others. Satisfaction or dissatisfaction in a supporting realm spills over into a superior domain. In the final analysis, since life is the most superior of the domains, it ends up being at the receiving end (Lee et al., 2001).

#### 2.1.2 Conflict theory

According to the conflict theory, what makes one fulfilled and gives a sense of achievement in one aspect of life is a consequence of some sacrifice in another aspect of life because there is an assumption that the two domains such as life and work are fundamentally incompatible with each other owing to inherent different norms and requirements. Work-life conflict is “a form of inter-role conflict in which the pressures of the role from the work and family domains are mutually non-harmonious in some respect” (Greenhaus and Beutell, 1985). This means that assuming one role becomes more difficult as a result of one’s involvement in other roles. The role theory is the foundation of conflict theory (Powell and Greenhaus, 2009). The scarcity perspective defines the role theory owing to the limited amount of time and energy for individuals to fulfill the obligations of their roles. The scarcity perspective explains the limited quantity of time and energy at the disposal of individuals which the various roles may share. Three conflict categories abound based on time, stress and behavior (Powell and Greenhaus, 2009). Limited time at the worker’s disposal, which constrains his ability to manage the demands of different roles effectively, is the cause of time-based conflicts. When individuals experience irregular shift work and work time not being flexible, it may cause stress-based conflict. Psychological demands of work, interaction fatigue and job burnout are the major causes of strain-based conflict. Lastly, behavior based conflicts are consequences of the exhibition of uncomplimentary behaviors by the demands of the work. Often, such work demands behaviors are not conducive to an individual’s family role and attempting to switch between the two roles becomes a source of conflict (Roy, 2016).

### 2.2 Empirical review and hypotheses development

Gragnano et al. (2020) investigated Work–life balance in relation to the importance of work–family and work–health balance and found that that health is as important as family in the WLB. Susanto et al. (2022) investigated the influence of “WLB and JS, on the job performance of the employees of SMEs with family-supportive supervisor behaviors as the moderator. They found that WLB has a positive influence on JS and performance and JS has a partial mediating influence on the relationship between WLB and job performance. Mensah and Adjei (2020) examined WLB the influence of self-reported health among working adults in Europe. They found that work-life conflict had a statistically significant relationship with poor self-reported health among working adults in Europe.

Lunau et al. (2019) studied the influence of Work–life balance and health on well-being in European welfare states. They found that workers compare health is as important as family in the WLB and more of the variation in JS is explained by work-health balance than WLB. Based on the foregoing and the negative spill-over effect of the spill-over theory, the study tested the following null hypotheses:

H_0_1: Devotion to family and employee has no significant influence on employees’ commitment to work.

H_0_2: Devotion to religion has no significant influence on employee’s commitment to work.

H_0_3: Observance of leisure has no significant influence on EMPC to work.

H_0_4: Implementation of health practices have no significant influence on the EMPC.

Delecta (2011) examined WLB from the organizational context and found a statistically significant correlation between long working hours occupational stress. Umma and Zahana (2020) investigated the determinants of WLB among teachers of a government school in Sri Lanka and found a significant negative correlation between workload/childcare and WLB; Otuya and Andeyo (2020) reviewed WLB and conclude that WLB is worth implementing by policymakers. Wijaya and Suwandana (2022) examined the influence of JS and WLB on the job performance of female Nurses to analyze the influence of JS and WLB on their Job Performance and found that WLB has a positive and significant effect on the performance of female nurses. Shouman et al. (2022) investigated WLB and firm innovativeness based on male and female bosses’ strategies. He found that employee WLB has no statistically significant influence on organizational performance.

Abdullah et al. (2022) studied WLB and JS in Chhattisgarh. In addition to the above Berglund et al. (2021) studied the influence of WLB on the predicted work ability of employees in Sweden. Thilagavathy and Geetha (2023) systematically reviewed literature on WLB, Tamunomiebi and Oyibo (2020) systematically reviewed WLB and employee performance and concluded that systemic barriers are hindering implementation of the policies on WLB in Nigeria. Lastly, Beauregard and Lesley (2009) examined how WLB practices relate to organizational performance and concluded that the use of WLB practices would attract individuals to an organization and significantly improve employee attitudes. Given the foregoing, the study tested the following null hypotheses:

Akinlade and Nwaodica (2021) examined work life balance and job satisfaction of employees in an international airport using a mixed method design/. They found that work stress affects the home and quality family life of the employees. Nadhiya and Sareena Umma (2022) studied work-life balance and job satisfaction among academics in Sri Lanka using a cross-sectional survey design. Their findings suggest that work-life balance stimulates job satisfaction among employees. Ethelmary and Nebolisa (2019) investigated work life balance and job satisfaction of selected commercial banks in Nigeria. They used the efforts recovery model as their framework and used a cross-sectional survey design. They analyzed the research data with correlation technique regression techniques. The results revealed that work load pressure, role conflict and family have statistically significant effects on job satisfaction. Babatunde et al. (2020) investigated Work life balance and the performance of academic staff tertiary institutions. The study used a cross-sectional survey design. They found that work flexibility and work environment which served as the indicators of work life balance significantly affect the employees’ performance. Mandagi and Wijono (2023) examined work-Life Balance and job satisfaction of employees using a cross-sectional survey design. They employed Pearson correlation in data analysis. The results of the correlation analysis revealed a strong association between work-life balance and job satisfaction.

Most of the empirical studies used employed review techniques. Apart from the studies of Mas-Machuca et al. (2016), Berglund et al. (2021), Abdullah et al. (2022), Wijaya and Suwandana (2022), and Boakye et al. (2023) that used good inferential test most, of the studies are either reviews of empirical studies or they used weak inferential statistics or inappropriate inferential tests. One of the studies used Chi-square statistic to test for the effect of work-life balance on employee commitment. Babatunde et al. (2020), Akinlade and Nwaodica (2021) as well as Nadhiya and Sareena Umma (2022), used descriptive technique in analyzing their data while Mandagi and Wijono (2023) used Pearson correlation technique to test for significance of data. These tend to cast some measure of doubts on the inferences associated with some of the empirical studies. Two important aspects of life activities ignored by empirical studies are *Religion* (Delecta, 2011; Mas-Machuca et al., 2016; Hsu et al., 2019; Lunau et al., 2019; Gragnano et al., 2020; Mensah and Adjei, 2020; Otuya and Andeyo, 2020; Khateeb, 2021; Abdullah et al., 2022; Wijaya and Suwandana, 2022; Boakye et al., 2023; Thilagavathy and Geetha, 2023), Leisure (Delecta, 2011; Lunau et al., 2019; Mensah and Adjei, 2020; Otuya and Andeyo, 2020; Susanto et al., 2022; Wijaya and Suwandana, 2022), Family Lunau et al. (2019). The relegation of these two aspects of life activities suggests that these factors are not considered important in the authors’ countries. However, religion and leisure are critical to life in Nigeria and many tribes, including the major tribes accord the two factors priority importance. To this end, a study of work-life balance that neglects religion and leisure is incomplete.

H_0_5: Employee JS does not have any significant influence on work commitment.

H_0_6: Employee JS does not significantly mediate the influence of WLB on EMPC.

## 3 Methodology

The study employed the quantitative research method. The research design is a survey of 344 employees from work communities in South–South and South-West geopolitical regions in Nigeria.

### 3.1 Population of the study

Employees consisting of academic and other staff of private and public Universities were the target population. Specifically, the study selected two Universities from each of the categories as well as employees of four multinational companies in South-west and South–South Nigeria constituted the study population.

### 3.2 Sampling framework

The study used Yamane formula in determining the sample size. The Taro Yamane formula took cognisance of a 5 % (5%) margin of error and 4,850 as the total number of employees investigated in the organizations under consideration. Consequently, this resulted in 370 as the sample size of the study shared this 370 to all the work communities proportionately. The researcher sought and elicited all relevant information about the employees from the appropriate authorities of the organizations and used stratified random sampling in selecting respondents for the study. Staff statuses served as the stratification criterion while the lottery technique served as the randomization technique. Based on the sample size, the study requested 370 participants but 344 of them consented. Consequently, the responses of the 344 respondents served as the input to the data analysis. The authors elicited the desired responses with a structured questionnaire which consisted of two sections. The two sections are the demographic section, which consists of the items on the respondents’ biodata, and the section on the items that addressed the research questions with a question-response format of the Likert Scale type.

### 3.3 Method of data collection

The study administered the research instrument to the respondents physically in their work settings and retrieved same physically using the services of *ad-hoc* research assistants. The author chose this method of data collection because of the need to minimize the rate of default coupled with the desire to seek the consent of the participants to participate in the study. This explains why the response rate was as high as 93 % (03%).

### 3.4 Measurement of variables

The study used one dependent variable (EMPC), one mediating variable (JS) and four independent variables (family, religion, leisure and health). The study used four five-point Likert-scale items to measure three of the independent variables and used five Likert-scale items to measure health. In addition, four Likert-scale items served to measure the mediating variable while five Likert scale items measured the dependent variable.

### 3.5 Validity and reliability

The study tested the questionnaire for validity and reliability to ensure that the data are usable for the purpose.

#### 3.5.1 Validity

The authors used a pilot study of 40 employees from work communities other than the study’s population. The responses elicited from respondents in the pilot test served to compute the study’s convergent and discriminant validities. The study conducted factor analysis from the data and the loadings served to compute the average variance extracted (AVE). The AVE served as a critical component in the computation of the convergent and discriminant validities. The Cut-off of the AVEs for convergent validity is 0.5 (Hair et al., 2009; Wei and Lu, 2013; Cheung S. et al., 2023). All the AVEs computed in the study are more than 0.5, thus establishing convergent validity (See Table 1).

**Table 1:** Convergent validity.

Variable		Convergent validity (AVE)
fml	=	0.803
rel	=	0.765
.lei	=	0.502
.hea	=	0.510
.js	=	0.617
.empc	=	0.780

**Figure 1:**
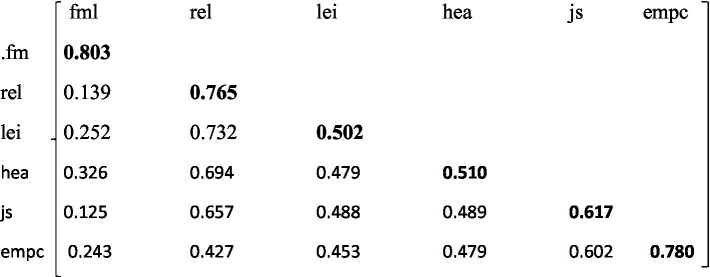
Discriminant validity.

After determining the convergent validity, the author also computed the discriminant validity, which requires the computation of the correlation coefficients. The study calculated the correlations and compared them with the square roots of the AVEs. The two validities help to validate a measuring instrument because the convergent validity helps to indicate the extent to which the measure sufficiently corresponds to measures of related constructs. The computed divergent validity indicates the degree to which the items are unrelated or negatively related to measures of distinct constructs. The results show that all the AVEs in the main diagonal are more than the corresponding correlation coefficients below the diagonals. To this end, discriminant validity is established (See Table 1; Figure 1).

#### 3.5.2 Reliability


$$\mathrm{Composite}\;\mathrm{Reliability}:\frac{{\left(\sum \lambda_{i}\right)}^{2}}{{\left(\sum \lambda_{i}\right)}^{2}+\sum e_{i}}$$

Where:

. $\lambda_{i}$ = factor Loadings (Standardized factor loadings.

.$e_{i}$ = 1 - ${\lambda_{i}}^{2.}$

All the computed values of the composite reliabilities are more than 0.5 for family (fml), religion (rel), leisure (rel), Health (hea), JS (js), and EMPC (emp) respectively, thus establishing composite reliability for all the constructs. The implication is that the research instrument is reliable and internally consistent (See Table 2).

**Table 2:** Composite reliability.

Variable		Composite reliability index
fml	=	0.945
rel	=	0.924
.lei	=	0.797
.hea	=	0.847
.js	=	0.877
.empc	=	0.959

### 3.6 Data analysis technique

The authors used Statistical Package for Social Sciences (SPSS) to code the data. The study used collinearity diagnostics technique to test for multicolinearity. The results indicate that all the variance inflation factors are less than 10, thus suggesting the absence of common method bias (See Table 3). Next, they inputted the data into STATA and analyzed the same using descriptive statistics. Subsequently, they used path diagram analysis of SEM in analysing the data. The results of the path diagram analysis formed the basis for the determination of the perceived WLB predictors and the influence of WLB on EMPC.

**Table 3:** Multicolliniarity tests.

		Volinearity diagnistics	
Model	Index	Tolerance limit	VIF
Constant			
.fml		0.77	1.306
.rel		0.27	3.703
.lei		0.101	6.878
hea		0.221	4.519
.js		0.168	4.956

*Mathematical specification of the Models*

The study specifies the models as:

.js = f(fml, rel, lei, hea) … (i)

Specifically,

.js = $\theta_{0}$ +$\theta_{1}$fml + $\theta_{2}$ rel + $\theta_{3}$ lei + $\theta_{4}$hea + e … (ii)

.empc = f (js,fml, rel, lei, hea) … (iii)

.empc = $\emptyset_{0}$ + $\emptyset_{1}$js + $\emptyset_{2}$ fml + $\emptyset_{3}$ rel + $\emptyset_{4}$lei + $\emptyset_{6}$ hea + e … (iv)

Where

.empc = EMPC

.js = JS

.fml = family

.rel = religion

.lei = leisure *sampling framework*.

.hea = health

.$\theta_{0}$ = proportion of the changes in JS that WLB does not account for

.$\theta_{i}$ (I = 1, 2 … 4) = proportion of the changes in JS that WLB accounts for

.$\emptyset_{0}$ = proportion of the changes in EMPC that WLB does not account for

.$\emptyset_{i}$ (I = 1, 2 … 5) = proportion of the changes in EMPC that WLB accounts for

### 3.7 Ethical statement

The study sought and got ethical approval from the Ethical Board of Studies of the corresponding author’s institution. However, the ethical approval is without an Ethical Approval Number as the Ethical Board is yet to complete the process of licensing. The authors complied with ethical requirements in the conduct of the study.

## 4 Results

### 4.1 Descriptive statistics

The study presents the descriptive statistics of the study, which consists of mean and standard deviations of the study’s variables as the descriptive statistics. The results indicate that all the mean values are higher than the cut-off value of 3. I. The results further indicate that the highest mean of 3.57 is for family while the least mean of 3.27 is for leisure. This portrays leisure as the least prioritized factor by the respondents. In addition, the results indicate that all the standard deviations are lower than 0.83, thus indicating that the data are not highly dispersed. The least standard deviation among the independents variables is for health while the highest is for leisure and religion, thus suggesting that respondent’s perception of health is the least dispersed and thus, most reliable of all the independent variables.

### 4.2 Inferential statistics

The results of the structural equation model of WLB and JS (direct effects) reveals that the coefficients of the individuals’ commitments to family, religion, leisure and health are −0.1839, −0.2651, 0.8160 and 0.4406, respectively. This means that a unit variation in dedication to family leads to 18.39% decrease in commitment to work and vice versa, a unit increase in the commitment to religion will cause a 26.51% decrease in the commitment to health and vice versa. In addition, a unit increase in the commitment to leisure will lead to an 81.6% increase in the commitment to work and vice versa while a unit increase in the commitment to health will lead to a 44.1% increase in the commitment to work and vice versa (See Tables 4, 5). Furthermore, the computed z statistics and their associated significant probabilities indicate that all the coefficients are statistically significant at the 1 % (1%) level. The implication is that we reject all the null hypotheses since the significant probabilities are all less than 0.01 (See Tables 4, 5). Thus, WLB significantly influences JS. While some aspects of life’s imbalance (family and health) have statistically significant negative influences on work, some other aspects of life’s imbalance (leisure and health) have statistically significant positive influences on work.

**Table 4:** Structural equation model of work-life balance and employee commitment with job satisfaction as a mediating variable (Direct effects).

	Coefficient	Standard error	*z*	*p* > |z|
Structural				
.js < − |				
.fml|	−0.1839	0.0260	−7.07	0.000
.rel|	−0.2651	0.0406	−6.53	0.000
.lei |	0.8160	0.0541	15.08	0.000
.hea|	0.4406	0.0490	9.00	0.000
.empc <− |				
.js |	0.4182	0.0394	10.61	0.000
.fml	0.0273	0.0204	1.34	0.180
.rel	−0.4417	0.0314	−14.05	0.699
.lei |	0.4033	0.0510	7.91	0.000
.hea|	0.2544	0.0398	6.40	0.000

**Table 5:** Structural equation model of work-life balance and employee commitment with job satisfaction as a mediating variable (Indirect effects)

	Coefficient	Standard error	*z*	*p* > |z|
Structural				
.js < −	|			
.fml|	0 (No path)			
.rel|	0 (No path)			
.lei |	0 (No path)			
.hea|	0 (No path)			
.empc <−	|			
.js |	0 (No path)			
.fml	−0.0769	0.0131	−5.88	0.000
.rel	−0.1109	0.0199	−5.56	0.000
.lei |	0.3413	0.0393	8.68	0.000
.hea|	0.1843	0.0268	6.56	0.000

The results of the structural equation model of WLB and EMPC (direct effects) reveal that the coefficients of JS, family, religion, leisure and health are 0.4182, 0.027, −0.4417, 0.4033 and 0.2544, respectively. This shows that a unit variation in JS will cause a − 41.82% change in EMPC, a unit change in dedication to family leads to 2.7% variation in EMPC, a unit change in the commitment to religion will cause a − 41.17% change in EMPC. In addition, a unit variation in the commitment to leisure will cause a 40.33% variation in EMPC, while a unit change in the commitment to health causes a 25.44% variation in EMPC (See Tables 4, 5). Furthermore, the computed z statistics and their associated significant probabilities indicate that all the coefficients of JS, religion, leisure and health are statistically significant at the 1 % (1%) level. The implication is that we reject all the associated null hypotheses since the significant probabilities are all less than 0.01 (See Tables 4, 5). However, the coefficient of the family proves to be insignificant as the *p*-value is 0.180. This means that dedication to family does not have any significant influence on EMPC. Thus, all the indicators used in measuring WLB, except family, have direct significant influence on EMPC with religion alone having a negative influence on EMPC while the remaining indicators (JS, leisure and health) have statistically significant positive influences on work (see Tables 4, 5).

The results of the structural equation model in testing WLB and EMPC (indirect effects) with the mediation of JS reveal that the coefficients of JS, family, religion, leisure and health are 0, −0.0769, −0.1109, 0.34013 and 0.1828, respectively. Thus indicating the absence of an indirect path from JS to EMPC. The results also indicate that a unit change in dedication to family leads to a − 7.69% variation in EMPC to work, and a unit variation in the commitment to religion will cause a − 11.09% variation in EMPC. In addition, a unit variation in the devotion to leisure will cause a 34.01% variation in EMPC. In addition, a unit change in the commitment to health will cause a 40.33% change in EMPC (See Tables 4, 5).

Furthermore, the computed z statistics and the associated significant probabilities reveal that all the coefficients of religion, leisure and health are statistically significant at the 1 % (1%) level. The implication is that we reject all the associated null hypotheses since the significant probabilities are all less than 0.01 (Tables 4, 5). Thus, all the indicators of WLB have significant influences on EMPC with JS as a mediator. Family and religion have statistically significant negative influences on EMPC while the other two indicators (leisure and health) have statistically significant positive influences on EMPC (Tables 4, 5).

The study used Root Mean Square Error (RMSE), Equation level goodness of fit test, Wald test and the fit Statistic to test for goodness of fit. The RMSE is approximately 0.2460, this is not too far from zero thus, indicating a good fit (See Table 6). Results of equation level goodness of fit test indicate that 0.6718 and 0.5605 are the fitted and predicted values of JS respectively, resulting in a residual value of 0.1113. Similarly, 0.4038 and 0.3444 are the fitted and predicted values of EMPC respectively, resulting in a residual value of 0.0594 (See Table 6). The small values of the residual values of JS and EMPC indicate a high predictive power of each model. These resulted in an overall goodness of fit of 0.9057. This means that 90.57% of the variation in EMPC results from the changes associated with JS and the indicators of WLB (family, religious activities, leisure, and health; Table 7).

**Table 6:** Goodness of fit tests.

Equation-level goodness of fit						
		|	Variance			
Depvars|	Fitted	Predicted	Residual	R-Squared	mc	mc^2^
Observed						
.js |	0.6718	0.5605	0.1113	0.8344	0.9134	0.8344
.empc |	0.4038	0.3444	0.0594	0.8528	0.9235	0.8528
Overall |	0.9057			RMSE	0.24596	
Wald’s teat						
	$Chi^{2}$		df.			*p*
Observed|						
*js|	173.28		4			0.000
empc|	199.32		5			0.000
Fit statistic			Value			Description
	+					
Likelihood ratio			0.000			
chi^2^_ms(0)						model vs. saturated
*p* > chi^2^						
chi2_bs(9)			1277.613			baseline vs. saturated
*p* > chi^2^			0.000			

**Table 7:** Summary of hypotheses.

Hypothesis	Comment
1, Devotion to family and employee has no significant influence on employees’ commitment to work	Reject
2. Devotion to religion has no significant influence on EMPC	Reject
3. Observance of leisure has no significant influence on EMPC to work	Reject
4. Implementation of health practices have no significant influence on the EMPC.	Reject
5. Employee JS does not have any significant influence on EMPWC	Reject
6. Employee JS does not significantly mediate the influence of WLB on EMPC	Reject

### 4.3 Discussion of findings

The study’s first hypothesis is “devotion to family and employee has no significant influence on employees’ commitment to work.” With a significant *p* < 0.01, the study rejects this hypothesis, thus implying that there is a statistically significant relationship between work-life balance and employee commitment through the mediation of job satisfaction. The results provide support to the studies of Mensah and Adjei, 2020, Abdullah et al. (2022) Wijaya and Suwandana (2022), Thilagavathy and Geetha (2023), and Boakye et al. (2023) on how family life interferes with work life,. The second hypothesis “devotion to religion has no significant influence on employee’s commitment to work” had a significant *p* < 0.01, and this led to the rejection of the hypothesis. The implication is that religion has a statistically significant influence on employee commitment through the mediation of job satisfaction. The results provide support for the studies of Lunau et al. (2019), Mensah and Adjei, 2020, Otuya and Andeyo, 2020, Susanto et al. (2022), and Wijaya and Suwandana, 2022, among others. The second hypothesis “observance of leisure has no significant influence on employee’s commitment to work” had a significant *p* < 0.01, and this led to the rejection of the hypothesis. The implication is that leisure has a statistically significant influence on employee commitment through the mediation of job satisfaction.

The fourth hypothesis “implementation of health practices have no significant influence on employee’s commitment to work” had a significant *p* < 0.01, and this led to the rejection of the hypothesis. The implication is that implementation of health practices have a statistically significant influence on employee commitment through the mediation of job satisfaction.

The results are consistent with Hsu et al. (2019), Gragnano et al. (2020), Mensah and Adjei, 2020, Otuya and Andeyo (2020), Khateeb (2021), Abdullah et al. (2022), Wijaya and Suwandana, 2022, Boakye et al. (2023), and Thilagavathy and Geetha (2023).

The fifth and sixth hypotheses are: “employee JS does not have any significant influence on work commitment” and “employee JS does not significantly mediate the influence of WLB on EMPC” respectively. The study rejected the two null hypotheses based on the p < 0.01 values. Thus, job satisfaction has a statistically significant relationship with employee commitment and job satisfaction has a statistically significant mediating effect on the relationship between the indicators of work-life balance (religion, family, health, leisure) and employee commitment.

The study’s test of hypotheses showed that work-life imbalance has significant influences on JS with family and religion having statistically significant negative influences on JS while leisure and health have statistically significant positive influences on JS. The implication is that the time spent on family and religion tends to deplete the work time and thus makes it have an inverse relationship with work. Thus, a disequilibrium in the apportionment of priority between work and religion will lead to dissatisfaction. In other words, if the individual spends more time at home and religion than at work, he will have dissatisfaction with the work but will be satisfied with family life and religious life and if he spends more time at work than home and religion he will be satisfied with the work but dissatisfied with family and religious lives. In this case, striking a balance between the times spent at work, home and religion is the only way he can get optimum satisfaction. The observed positive (direct) relationship between leisure and JS as well as health and JS suggests that both are complementary to JS. In other words, as the time an individual spends on leisure increases, the more relaxed he becomes and the more prepared and satisfied with the job he becomes. In the same vein, the more an individual complies with health practices, the healthier he becomes and the more satisfied he becomes, as he is equipped with the mental and physical capacity to discharge his duties. These results agree with, Hsu et al. (2019), Lunau et al. (2019), Gragnano et al. (2020), Mensah and Adjei (2020), Abdullah et al. (2022), Susanto et al. (2022), and Wijaya and Suwandana (2022).

In comparing employee life domain with work commitment using family, religion, leisure and health as life domain, the results reveal that family has a positive but insignificant direct relationship with EMPC while employee devotion to religion has a negative and significant direct relationship between and commitment to work. In addition, dedication to leisure and health consciousness have direct positive and statistically significant influences on an individual’s commitment to work.

An indirect test of how employee life domain relates with commitment to work using the mediation of JS reveals that the mediation was statistically significant as all the indicators of life (family, religion, leisure and health) had significant influences on EMPC to work, including family that was insignificant in the direct relationship. The implication is that there is a full mediation effect of JS on the relationship between family and EMPC while it had partial mediation effects on the relationships between the other indicators (religion, leisure and health) and EMPC. It is further pertinent to note that family and religion had statistically significant negative influences on work commitment while the influences of leisure and health on work commitment are statistically significant and positive. The results are consistent with Hsu et al. (2019), Lunau et al. (2019), Gragnano et al. (2020), Mensah and Adjei (2020), Abdullah et al. (2022), Susanto et al. (2022), and Wijaya and Suwandana (2022).

### 4.4 Proposed model of work-life and EMPC

**Figure 2:**
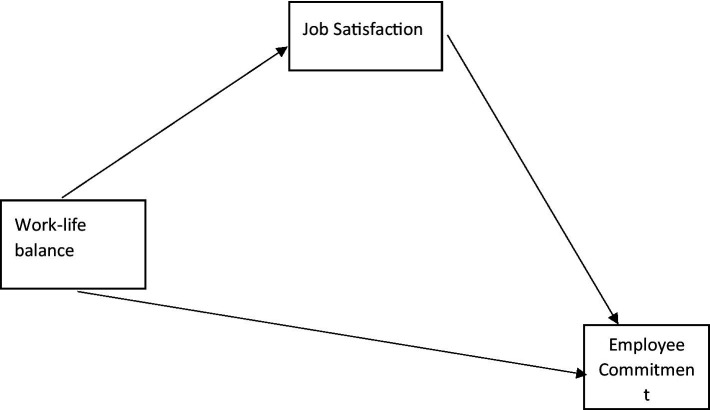
Proposed model of work-life and employee commitment.

In view of the findings, the study proposes a model that relates work-life to EMPCs. Equations 1–3 present the mathematical specifications of the models (See Figure 2). The models indicate that devotion to family does not have a direct relationship with EMPC but has an indirect relationship through the mediation of JS, thus suggesting a full mediation effect. Religion, leisure and health have direct as well as mediated relationships with EMPC, thus suggesting partial mediation effects.

.js = f(fml, rel, lei, hea) … (1)

.empc = f(rel, lei, hea) … (2)

.empc = f (js,fml, rel, lei, hea) … (3)

### 4.5 Theoretical contributions

This study has made some theoretical contributions. The spill-over has positive and negative effects. The positive spill-over assumes that the fulfillment and attainment that accrue as benefits from domain are a consequence of positive experiences in another realm while the negative spill-over states that work and non-work spheres relate inversely and antithetically. The significant positive associations which leisure has with JS/EMPCs as well as that which health has with JS/EMPC are supportive of the positive spill-over effects in the spill-over theory, as the positive experiences at work tend to reflect the positive experiences of leisure and health compliance. Nevertheless, the significant negative relationships which family has with JS/EMPC and that which religion has with JS/EMPC are supportive of inverse and antithetic relationships that exist between work and non-work domains. The results also supports the conflict theory since the results portray employee JS as a consequence of their sacrifice in family and religious commitment and vice versa since the resultant pressures arising from the employee’s work role and the employees’ commitment to family domains (religious and family activities) are mutually non-harmonious in some respect. In a nutshell, the study makes significant contributions to the positive and negative effects of the spill over theory.

### 4.6 Practical/policy implications

Given that disequilibrium in individuals’ work-life can influence JS/EMPC and ultimately the achievement of organizational goals, the need for strategic managers to formulate policies that will assist their employees in minimizing work-life disequilibrium becomes expedient. The reason is that by assisting the employees to minimize work-life disequilibrium, they are invariably contributing to their JS/commitment and ultimately to the organizations’ productivity. The strategic managers of firms and policymakers in government establishments that want to get the best from their employees can formulate policies to ensure that employee workload is within the capacity of the employees to enable them to discharge their duties within a reasonable time during the official hours. As much as possible, they should avoid work overload. Management should also sensitize their employees from time to time on the need to imbibe the habit of relaxing during their leisure hours rather than engaging in stressful non-work activities. Most importantly, management should encourage employees to be health conscious through good feeding habits and regular check-ups to enable them to stay healthy. Policymakers and strategic managers can also organize seminars to enlighten their employees on the need to deliberately strategize on striking a balance between work life and family life to facilitate fulfillment and healthiness. Useful insights on how a work-life balance can assist employees will stimulate their commitment to its goals

## 5 Conclusion

In conclusion, we reiterate the question; for what reason do we work? Is it to live? Why do we live? Is it to work”? It is pertinent to note that we both work to live and live to work. If we spend all our time working, where is the living? If we spend all our time living, where is the working for sustenance? We achieve sustainability when we strike a balance between work-life, and thus, the WLB. The study investigated WLB and EMPC with the mediation of JS. A survey of 344 randomly selected respondents from work communities in Nigeria presented the data and path diagram analysis of SEM served to analyze the data. Findings of the study show that there is a positive and significant relationship between JS and EMPC. In addition, family and religion significantly relates with EMPC negatively through the mediation of JS while leisure and health have statistically significant positive relationships with EMPC with JS as the mediating variable.

The study has made theoretical contributions to the spill-over and conflict theories through the negative relationships between family and EMPC as well as religion and EMPC, which are supportive of the negative aspect of the spill-over theory and the conflict theory. In addition, the significance of the relationships between leisure/health and EMPC which are supportive of the positive spill-over theory and the conflict theory. The study has also made an empirical contribution being about the only one to have so far examined how WLB influences EMPC as well as being the first to have incorporated family, religion, leisure and health in a single framework to examine the influence of WLB on EMPC.

The study encountered some constraints that suggest the need for further studies on the research problem. The first limitation concerns the restriction of the respondents to several indicators as is consistent with quantitative studies. Future studies should attempt to include open-ended questions and use a mix-method consisting of quantitative and qualitative techniques. Secondly, although the sample size is large enough, considering the diversity of Nigeria, future studies should try to make the sample more diverse by considering and including other regions before determining the sample size.

## Data availability statement

The raw data supporting the conclusions of this article will be made available by the authors, without undue reservation.

## Ethics statement

Written informed consent was obtained from the individual(s) for the publication of any potentially identifiable images or data included in this article.

## Author contributions

HI: Conceptualization, Data curation, Formal analysis, Investigation, Methodology, Project administration, Resources, Supervision, Validation, Visualization, Writing – original draft, Writing – review & editing.

## Appendix

Part one demographic characteristics

1. Status of respondent: Management Staff [] Senior Staff [] Junior Staff []

2. Gender: Male [] Female []

3. Highest Educational Qualification: School Certificate [] First Degree [] Master’s Degree [] Ph. D []

Part Two: Instruction: Indicate the extent to which you agree with the following items

**Table:**

S/N	Item	1	2	3	4	5
A	Family					
1	I spend time with my family					
2	I pay attention to what goes on in my family					
3	I do not see family matters as a distraction					
4	I take my family out for recreations					
B	Religion					
5	I am committed to religious activities					
6	I cannot compromise my religious programs for anything					
7	Religion is critical to life success					
8	Religious activities a critical to human survival					
C	Leisure					
9	I devout dime to playing games					
10	I devout time to watching movies					
11	I take time out to hang out with friends to have some drinks					
12	I enjoy participating in picnics					
D	Health					
13	I go for check-ups regularly					
14	I am conscious of what I eat					
15	I practice self-care and					
16	I sleep adequately					
17	I exercise my body regularly by walking					
E	Job satisfaction					
18	My work is interesting					
19	I love the conditions of service in my organization					
20	I am satisfied with my remuneration					
21	I like my work environment					
F	Employee commitment					
22	I love my job					
23	I do not intend to change to another organization					
24	I do not want to lose my job					
25	My work means a lot to me					
26	I meet assignment deadlines					

## References

[ref1] Abdullah H. Kabia S. K. Pandey P. (2022). Impact of work life balance on JS: a study of Chhattisgarh. J. Posit. Sch. Psychol. 6, 126–135,

[ref2] Agha K. Azmi F. T. Irfan A. (2017). WLB and JS: an empirical study focusing on higher education teachers in Oman. Int. J. Soc. Sci. Hum. 7, 164–171. doi: 10.18178/ijssh.2017.V7.813

[ref3] Ahmad M. R. Raja R. (2021). Employee JS and business performance: the mediating role of organizational commitment. Vision 25, 168–179. doi: 10.1177/0972262920985949

[ref4] Akinlade O. C. Nwaodica C. A. (2021). Work life balance and job satisfaction of employees in Murtala Muhammed and victor Attah international airports in Nigeria. KIU J. Soc. Sci. 7, 111–120,

[ref5] Babatunde S. O. Olanipekun W. D. Lateef S. A. Babalola H. B. (2020). Work life balance and the performance of academic staff at the selected tertiary institutions in Kwara state, Nigeria 55, 45. doi: 10.35741/issn.0258-2724.55.6.45,

[ref6] Beauregard T. A. Lesley C. H. (2009). Making the link between WLB practices and organizational performance. Hum. Resour. Manag. Rev. 19, 9–22. doi: 10.1016/j.hrmr.2008.09.001

[ref7] Berglund E. Anderzén I. Andersén A. Lindberg P. (2021). Work-life balance predicted work ability two years later: a cohort study of employees in the Swedish energy and water sector. BMC Public Health 21:1212. doi: 10.1186/s12889-021-11235-4 34167506 PMC8223187

[ref8] Boakye A. N. Asravor R. K. Essuman J. (2023). WLB as predictors of JS in the tertiary educational sector. Cogent Bus. Manag. 10:1. doi: 10.1080/23311975.2022.2162686

[ref9] Bocean C. G. Popescu L. Varzaru A. A. Avram C. D. Iancu A. (2023). WLB and employee satisfaction during COVID-19 pandemic. Sustain. For. 15:11631. doi: 10.3390/su151511631

[ref10] Cheung G. W. Cooper-Thomas H. D. Lau R. S. Wang L. C. (2023). Reporting reliability, convergent and discriminant validity with structural equation modeling: a review and best-practice recommendations. Asia Pac. J. Manag. 2023:9871. doi: 10.1007/s10490-023-09871-

[ref11] Cheung S. Elias R. Xie P. Rosenwaks Z. Palermo G. D. (2023). A non-randomized clinical trial to determine the safety and efficacy of a novel sperm sex selection technique. PLoS One 18:e0282216. doi: 10.1371/journal.pone.0282216, PMID: 36947521 PMC10032484

[ref12] Delecta P. (2011). Work life balance. Int. J. Curr. Res. 33, 186–189,

[ref13] Ethelmary D. Nebolisa O. T. (2019). Work life balance and job satisfaction of selected commercial banks in south-East Nigeria. Int. J. Entreprep. Bus. Innov. 2, 63–76,

[ref14] Gragnano A. Simbula S. Miglioretti M. (2020). Work–life balance: weighing the importance of work–family and work–health balance. Int. J. Environ. Res. Public Health 17, 1–20. doi: 10.3390/ijerph17030907 PMC7037206 32024155

[ref9008] Greenhaus J. H. Beutell N. J. (1985). Sources and conflict between work and family roles. Acad. Manag. Rev. 10, 76–88. doi: 10.2307/258214

[ref9007] Greenhaus J. H. Collins K. M. Shaw J. D. (2003). The relation between work–family balance and quality of life. J. Vocat. Behav. 63, 510–531. doi: 10.1016/S0001-8791(02)00042-8

[ref15] Gupta R. Agrawal R. (2023). Unveiling the hidden layers of employees’ JS and organizational commitment: a meta-analysis. Bus. Perspect. Res. 2023:11488. doi: 10.1177/22785337221148885

[ref16] Hair J. F. Black W. C. Babin B. J. Anderson R. E. (2009). Multivariate Data Analysis. 7th Edn. Upper Saddle River: Prentice Hall, 761.

[ref17] Hsu Y. Bai C. Yang C. Huang Y. Lin T. Lin C. (2019). Long hours’ effects on WLB and satisfaction. Biomed. Res. Int. 2019:8. doi: 10.1155/2019/5046934, PMID: 31341900 PMC6612405

[ref18] Khateeb F. R. (2021). Work life balance-a review of theories, definitions and policies. Cross Cult. Manag. J. 23, 27–55.

[ref9001] Kirchmeyer C. (2000). “Work -life initiatives: greed or benevolence regarding workers’ time?” in Trends in organizational behavior: time in organizational behavior. eds. Cooper C. L. Rousseau D. M. (Chichester: Wiley), 7, 79–9.

[ref9002] Lee M. H. Lee H. J. Ryu P. D. (2001). Public health risks: Chemical and antibiotic residues review. Asian-Australas J. Anim. Sci. 14, 402–413.

[ref19] Lunau T. Bambra C. Eikemo T. A. van der We K. A. Dragano A. (2019). A balancing act? Work–life balance, health and well-being in European welfare states. Int. J. Environ. Res. Public Health 17, 422–420. doi: 10.1093/eurpub/cku010 24567294

[ref20] Mandagi M. M. Wijono S. (2023). Work-life balance (WLB) and job satisfaction of employees at PT. X Yogyakarta. J. Soc. Res. 2, 2557–2563. doi: 10.55324/josr.v2i8.1301

[ref21] Mas-Machuca M. Berbegal-Mirabent J. Alegre I. (2016). WLB and its relationship with organizational pride and JS. J. Manag. Psychol. 31, 586–602. doi: 10.1108/JMP-09-2014-0272

[ref22] Mensah A. Adjei N. K. (2020). WLB and self-reported health among working adults in Europe: a gender and welfare state regime comparative analysis. BMC Public Health 20:1052. doi: 10.1186/s12889-020-09139-w, PMID: 32669103 PMC7364652

[ref23] Nadhiya A. L. F. Sareena Umma M. A. G. (2022). Work-life balance and job satisfaction: Study among the academics of South Eastern University of Sri Lanka. Oluvil: South Eastern University of Sri Lanka.

[ref24] Otuya W. Andeyo L. M. (2020). WLB: a literature review. Strateg. J. Bus. Change 7, 249–258.

[ref9003] Parker S. R. Scott M. H. (1971). Developing models of workplace industrial relations. Br. J. Ind. Relat. 9, 214–224. doi: 10.1111/j.1467-8543.1971.tb008n6

[ref25] Pehlivanoğlu M. Eymür E. Civelek M. E. (2022). The relationship between JS and organizational commitment in female employees. J. Appl. Theor. Soc. Sci. 4, 406–422. doi: 10.37241/jatss.2022.74

[ref9004] Powell G. N. Greenhaus J. H. (2009). Sex, gender, and decisions at the family → work interface. J. Manag. doi: 10.1177/0149206309350774

[ref26] Qureshi M. A. Qureshi J. A. Thebo J. A. Shaikh G. M. Brohi N. A. Qaiser S.. (2019). The nexus of employee’s commitment, JS, and job performance: an analysis of FMCG industries of Pakistan. Cogent Bus. Manag. 6:1. doi: 10.1080/23311975.2019.1654189

[ref9005] Roy A. (2016). Who’ Afraid of Postcolonial Theory? Int. J. Urban Reg. Res. 40, 200–209. doi: 10.1111/1468-2427.12274

[ref27] Shouman L. Vidal-Suñé A. Alarcón A. (2022). Impact of WLB on firm innovativeness: the different strategies used by male and female bosses. Adm. Sci. 12:115. doi: 10.3390/admsci12030115

[ref9006] Staines G. L. (1980). Spillover versus compensation: A review of the literature on the relationship between work and Non-work. Human Relations 33, 111–129.

[ref28] Susanto P. Hoque M. E. Jannat T. Emely B. Zona M. A. Islam M. A. (2022). WLB, JS, and job performance of SMEs employees: the moderating role of family-supportive supervisor behaviors. Front. Psychol. 13:906876. doi: 10.3389/fpsyg.2022.906876, PMID: 35800926 PMC9253617

[ref29] Tamunomiebi M. D. Oyibo C. (2020). WLB and employee performance: a literature review. Eur. J. Bus. Manage. Res. 5:196. doi: 10.24018/ejbmr.2020.5.2.196

[ref30] Thilagavathy S. Geetha S. N. (2023). WLB –a systematic review. J. Manag. 20, 258–276. doi: 10.1108/XJM-10-2020-0186

[ref31] Umma S. M. A. Zahana F. M. M. (2020). Factors affecting the work life balance: study among the teachers of a government school in Sri Lanka. J. Manag. 15, 65–73. doi: 10.4038/jm.v15i2.7604

[ref9009] Vijayakumar G. Janakiram B. (2017). Theories of work-life balance – a conceptual review, Int. Res. J. Manag. Commerce. 4, 1–4.

[ref32] Wambui M. L. Cherotich B. C. Emily T. Dave B. (2017). Effects of work life balance on employees’ performance in institutions of higher learning. A case study of Kabarak university. Kabarak J. Res. Innov. 4, 60–79. doi: 10/j.foodcont.2017.05.036

[ref33] Wei P. S. Lu H. P. (2013). An examination of the celebrity endorsements and online customer reviews influence female consumers’ shopping behavior. Comput. Human Behav. 29, 193–201. doi: 10.1016/J.CHB.2012.08.005

[ref34] Wijaya P. D. G. K. Suwandana G. M. (2022). The role of JS, WLB on the job performance of female nurses at local general hospital. Eur. J. Bus. Manag. Res. 7, 208–212. doi: 10.24018/ejbmr.2022.7.1

